# The GspCD-dependent type II secretion system promotes necrotizing soft tissue infection caused by *Aeromonas hydrophila*

**DOI:** 10.3389/fcimb.2026.1870837

**Published:** 2026-06-30

**Authors:** Yuka Tonosaki, Kohei Yamazaki, Shota Owada, Kei Yamaguchi, Takashige Kashimoto

**Affiliations:** Laboratory of Veterinary Public Health, School of Veterinary Medicine, Kitasato University, Aomori, Japan

**Keywords:** *Aeromonas hydrophila*, extracellular protein secretion, necrotizing soft tissue infection, type II secretion system, virulence

## Abstract

Necrotizing soft tissue infections (NSTIs) are fulminant bacterial diseases characterized by rapid tissue destruction, systemic deterioration, and high mortality. *Aeromonas hydrophila* is an important causative agent of NSTIs, but the bacterial mechanisms that promote tissue destruction, *in vivo* expansion, dissemination, and host lethality remain incompletely understood. Here, we investigated the contribution of the GspCD-dependent type II secretion system (T2SS) to *A. hydrophila* virulence using transposon mutants, extracellular protein analyses, and a mouse NSTI model. Mutants carrying transposon insertions in *gspD* and *gspC* showed defective secretion of a FLAG-tagged truncated AerA construct and markedly reduced hemolytic activity in culture supernatants. Comparative analysis of extracellular proteins further showed that disruption of *gspC* reduced the abundance of multiple known or predicted virulence-associated extracellular factors, including AerA, Ahh, lipase, and metalloprotease. In the mouse NSTI model, both mutants exhibited attenuated virulence, including reduced serum markers of tissue injury, less severe histopathological damage, impaired *in vivo* expansion and dissemination, and decreased lethality. Together, these findings show that the GspCD-dependent T2SS plays an important role in *A. hydrophila* NSTI pathogenesis by promoting extracellular protein secretion, tissue destruction, bacterial spread, and host mortality.

## 1 Introduction

Necrotizing soft tissue infections (NSTIs) are rapidly progressive and life-threatening bacterial diseases characterized by extensive destruction of subcutaneous tissue and skeletal muscle, systemic deterioration, and high mortality (Stevens and Bryant, 2017; Peetermans et al., 2020). *Aeromonas hydrophila* is an important etiologic agent of NSTIs, particularly in wound-associated infections following exposure to aquatic environments (Lai et al., 2007; Devos et al., 2020; Fernandez-Bravo and Figueras, 2020; Janda and Abbott, 2010; Parker and Shaw, 2011). Clinical cases of *A. hydrophila* NSTIs are often fulminant and can rapidly progress to septic shock and death, underscoring the need to better understand the bacterial mechanisms that drive invasive soft tissue disease (Chao et al., 2013; Devos et al., 2020; Gold and Salit, 1993; Huang et al., 2021).

Several virulence factors have been implicated in the pathogenesis of *A. hydrophila*, including proteases, lipases, and extracellular toxins such as Aerolysin and hemolysin, many of which are secreted via the type II secretion system (T2SS) (Fernandez-Bravo and Figueras, 2020; Janda and Abbott, 2010; Tomas, 2012; Grim et al., 2014). Among these, Aerolysin has been extensively studied as a pore-forming toxin that damages host cell membranes and contributes to cytotoxicity (Abrami et al., 2000; Galindo et al., 2006; Parker et al., 1996; Iacovache et al., 2008). Earlier genetic studies also demonstrated that aerolysin deficiency attenuates *A. hydrophila* virulence in mice (Chakraborty et al., 1987). However, the bacterial mechanisms that coordinate tissue destruction, bacterial proliferation within infected tissues, dissemination, and lethality during NSTIs remain incompletely understood. We have previously shown that motility is required for efficient spread of *A. hydrophila* within soft tissues (Yamazaki et al., 2021), indicating that active bacterial dissemination is a key determinant of disease progression. Because this process is likely constrained by physical barriers such as the extracellular matrix and host cell integrity, we hypothesized that T2SS-dependent secretion of extracellular toxins and degradative enzymes promotes bacterial proliferation and dissemination by disrupting host tissues and removing these barriers. Thus, in addition to motility, secretion-dependent remodeling of the local tissue environment may be required for destructive and lethal NSTI progression.

The T2SS is a conserved secretion apparatus in Gram-negative bacteria that exports folded proteins from the periplasm to the extracellular milieu. T2SS substrates are first transported across the inner membrane by the Sec or Tat pathway and are then secreted across the outer membrane through a multiprotein complex containing the secretin GspD (Cianciotto, 2005; Potempa and Potempa, 2012; Green and Mecsas, 2016; Korotkov et al., 2012). GspC is thought to contribute to substrate recognition and to the functional linkage of the secretion machinery (Cianciotto, 2005; Potempa and Potempa, 2012; Green and Mecsas, 2016; Korotkov et al., 2012). In several Gram-negative pathogens, the T2SS is required for secretion of major virulence factors and hydrolytic enzymes, supporting a central role for this system in extracellular protein export and pathogenesis (Cianciotto, 2005; Reichow et al., 2010; Sikora, 2013; Ali et al., 2000). Despite the established importance of secreted toxins in *Aeromonas* pathogenesis, the contribution of the T2SS itself to necrotizing soft tissue infection has not been directly defined in a murine model.

In the present study, we performed phenotypic screening of a transposon mutant library of *A. hydrophila* and identified mutants carrying insertions in *gspD* and *gspC*. Using these mutants, we investigated how the GspCD-dependent T2SS contributes to extracellular protein secretion, hemolytic activity, tissue damage, *in vivo* expansion and dissemination, and host lethality in a mouse NSTI model. Our findings indicate that this secretion system drives severe soft tissue infection by coordinating the export of extracellular factors that promote tissue destruction, bacterial spread, and host death.

## 2 Materials and methods

### 2.1 Bacterial strains and culture conditions

*Aeromonas hydrophila* clinical isolate RIMD 111065 and its derivatives were used in this study. The strain was originally isolated from human blood and was obtained from the Pathogenic Microbial Resource Unit, Research Institute for Microbial Diseases, Osaka University. A spontaneous rifampicin-resistant derivative of the wild-type (WT) strain was used for transposon mutagenesis and animal infection experiments. Bacteria were routinely cultured in Luria-Bertani (LB) broth at 37 °C with shaking at 200 rpm or on LB agar plates. When required, chloramphenicol (5 µg/mL for *A. hydrophila* and 10 µg/mL for *Escherichia coli*) or kanamycin was added for plasmid maintenance or mutant selection.

For *in vivo* bioluminescence imaging, a bioluminescent derivative carrying the *luxCDABE* operon integrated into the chromosome was used. Chromosomal integration was achieved using a suicide vector pYAK1 (Yamazaki et al., 2021), carrying the *luxCDABE* cassette flanked by *flaA* homologous arms, followed by allelic exchange. This chromosomal integration enabled stable maintenance of the bioluminescent marker without antibiotic selection.

### 2.2 Plasmid construction and complementation

Genomic DNA from the WT strain was purified using the DNeasy Blood & Tissue Kit (Qiagen, Hilden, Germany). The *aerA*, *gspD*, and *gspC* genes were amplified by PCR using gene-specific primers.

For detection of Aerolysin secretion, a truncated *aerA* construct encoding the secretion signal and N-terminal region of AerA (612 bp; 204 amino acids) was amplified. A FLAG tag (8 amino acids) was fused to its C terminus, generating a construct with a predicted molecular mass of approximately 20 kDa. A truncated construct was used to avoid potential biological effects associated with expression of full-length Aerolysin.

The pACYC184 vector backbone was amplified by PCR and assembled with each insert using the NEBuilder HiFi DNA Assembly Master Mix (New England Biolabs, Ipswich, MA, USA). The resulting plasmids were transformed into *Escherichia coli* DH5α, and transformants were selected on LB agar plates containing chloramphenicol. Plasmids were then introduced into *A. hydrophila* mutant strains by conjugation, and complemented strains were selected on LB agar plates containing chloramphenicol.

### 2.3 Construction of a transposon mutant library

A transposon mutant library was generated as described previously with minor modifications (Yamazaki et al., 2026a, b). Briefly, *A. hydrophila* was statically cultured in LB broth at 25 °C for more than 12 h, harvested by centrifugation at 10,000 × *g* for 1 min, and washed three times with lactose broth. *E. coli* BW19795 carrying pUT mini-Tn5 Km2 Tag was statically cultured in LB broth containing kanamycin at 37 °C and washed in the same manner. The transposon was introduced into *A. hydrophila* by conjugation with BW19795, and transconjugants were selected on agar plates containing appropriate antibiotics.

### 2.4 Identification of transposon insertion sites

Transposon insertion sites were identified by arbitrarily primed PCR as described previously (Yamazaki et al., 2026a, b). Primer sequences used for arbitrarily primed PCR are listed in **Supplementary Table 1**. Briefly, genomic DNA was extracted from each transposon mutant and subjected to PCR using a transposon-specific primer together with arbitrary primers targeting the chromosome. PCR products were sequenced, and insertion sites were determined by sequence homology searches against the *A. hydrophila* genome sequence. In addition, whole-genome sequencing analysis of the mutant strains was performed to assess the presence of additional transposon insertions. Under the analysis conditions used in this study, no evidence of a second transposon insertion was detected in either mutant.

### 2.5 Quantification of colony opacity

To evaluate colony opacity, overnight cultures were spotted onto LB agar plates and incubated at 37 °C for 12 h. Colony images were captured under identical conditions, and colony opacity/translucency was quantified using ImageJ software as described previously (Guzmán et al., 2014; Yamazaki et al., 2019). Relative values were calculated by setting the WT value to 100. The assay was performed independently three times.

### 2.6 Analysis of secreted proteins by SDS-PAGE, silver staining, and western blotting

WT, OS8, and OS9 were cultured in 5 mL of LB broth at 37 °C with shaking overnight. Culture turbidity was measured at OD_600_, and each culture was adjusted to the same optical density. The normalized cultures were inoculated into fresh LB broth and incubated for an additional 3 h at 37 °C with shaking. After incubation, cultures were readjusted to OD_600_ = 1.0 and centrifuged at 10,000 rpm for 10 min at room temperature to separate culture supernatants and bacterial pellets. For preparation of secreted protein samples, culture supernatants were precipitated with trichloroacetic acid (TCA) on ice for 20 min and centrifuged at 12,000 rpm for 20 min at 4 °C. The resulting pellets were washed with cold diethyl ether, centrifuged again at 12,000 rpm for 20 min at 4 °C, air-dried, and resuspended in SDS sample buffer. Bacterial pellets were directly resuspended in SDS sample buffer. Samples were separated by SDS-PAGE using a molecular weight marker (FastGene™ BlueStar Prestained Protein Marker, Nippon Genetics, NE-MWP03) and visualized by silver staining using the Silver Stain II kit (FUJIFILM Wako Pure Chemical Corporation, Osaka, Japan).

For detection of FLAG-tagged AerA, prepared samples were separated by SDS-PAGE, transferred onto membranes, and probed with monoclonal ANTI-FLAG M2 antibody (Sigma-Aldrich, St. Louis, MO, USA).

### 2.7 Proteomic analysis of culture supernatants

Proteomic analysis was performed using culture supernatants from WT and OS9. For each strain, 6 mL of bacterial culture supernatant was submitted for comparative quantitative proteomic analysis. Briefly, 200 µL of each sample was mixed with PTS buffer containing 12 mM sodium deoxycholate, 12 mM sodium lauroyl sarcosinate, and 50 mM ammonium bicarbonate supplemented with Complete EDTA-free protease inhibitor. Samples were heat-inactivated at 95 °C for 5 min, sonicated, precipitated, and resuspended in PTS buffer. Proteins were then reduced with 500 mM tris(2-carboxyethyl)phosphine (TCEP) and alkylated with 500 mM iodoacetamide at 37 °C for 60 min. After purification with SeraMag Speed Beads and washing with 70% ethanol, proteins were digested overnight at 37 °C with Lys-C and trypsin. The resulting peptides were acidified with 20% trifluoroacetic acid, desalted using StageTips, dried in a SpeedVac, and resuspended in 10 µL of buffer containing 1% TFA and 2% acetonitrile.

Peptides were analyzed by nanoLC-MS/MS using a Q Exactive Plus mass spectrometer coupled to an UltiMate 3000 RSLCnano HPLC system and an HTC-PAL autosampler. Peptides were separated on an in-house packed reversed-phase column (ReproSil-Pur 120 C18-AQ, 1.9 µm resin, 300 mm × 75 µm) with an Acclaim C18 PepMap100 trap column. Mobile phase A consisted of 2% acetonitrile with 0.1% formic acid in water, and mobile phase B consisted of 90% acetonitrile with 0.1% formic acid in water. Peptides were eluted at a flow rate of 280 nL/min at 60 °C using a 60-min gradient. Mass spectrometry was performed in positive ion mode using data-independent acquisition (DIA). MS1 scans were acquired in the Orbitrap at a resolution of 140,000 with an AGC target of 3e6, a maximum injection time of 200 ms, and a scan range of m/z 398–802. DIA MS2 scans were acquired at a resolution of 35,000 with an AGC target of 3e6, isolation window of 12 m/z, fixed first mass of 200.0 m/z, and normalized collision energy of 27.

Raw data were analyzed using DIA-NN version 1.8.1 for protein identification and quantitative analysis against the UniProtKB *Aeromonas hydrophila* database together with a contaminant protein database. Trypsin/P was specified as the digestion enzyme with up to two missed cleavages. Carbamidomethylation of cysteine was set as a fixed modification, and N-terminal methionine excision was included as a variable modification. Precursor- and protein-level false discovery rates were set to <1%. Protein intensity values were calculated as log2-transformed quantitative values based on MaxLFQ after correction using the top 500 precursors. Proteins with reduced abundance in OS9 relative to WT were used for downstream analysis.

### 2.8 Hemolysis assay

For qualitative evaluation of hemolysis, bacterial strains were cultured in 5 mL of LB broth at 37 °C with shaking for 15 h. Cultures were adjusted to OD_600_ = 1.0, serially diluted 10-fold, and 1.0 µL of each dilution was spotted onto sheep blood agar plates. After incubation at 37 °C for at least 12 h, hemolytic zones were examined.

For quantitative hemolysis assays, bacterial cultures were prepared under the same conditions and separated into culture supernatants and bacterial suspensions. Mouse erythrocytes were prepared as a 2% suspension in phosphate-buffered saline (PBS) after careful removal of plasma and buffy coat. Aliquots (50 µL) of the erythrocyte suspension were dispensed into 96-well plates and mixed with 50 µL of either culture supernatant or bacterial suspension. After incubation at 37 °C for 30 min, plates were centrifuged at 2,400 rpm for 10 min, and the supernatants were transferred to new plates. Absorbance was measured at 540 nm. Complete hemolysis was defined using distilled water, and nonhemolytic controls were prepared using PBS. Hemolytic activity was expressed as a percentage relative to the complete hemolysis control.

### 2.9 Animals

Five-week-old female C57BL/6 and BALB/c mice were purchased from Japan Jackson Laboratory and housed under specific-pathogen-free conditions with free access to food and water on a 12-h light/dark cycle. C57BL/6 mice were used for all animal experiments except *in vivo* bioluminescence imaging, for which BALB/c mice were used. All animal experiments were approved by the Institutional Animal Care and Use Committee of Kitasato University and were performed in accordance with the guidelines of the Japanese Association for Laboratory Animal Science (JALAS) (approval no. 24-033). This study is reported in accordance with the ARRIVE guidelines.

### 2.10 Mouse NSTI model

Bacteria cultured at 37 °C were diluted 1:100 in fresh medium and incubated for an additional 8 h. Cultures were collected by centrifugation at 7,000 × *g* for 3 min, washed once with PBS, and resuspended in the same medium. Each mouse was inoculated subcutaneously in the right thigh with 1 × 10^7^ CFU in 50 µL (n = 6 per group unless otherwise indicated). Mice were anesthetized with isoflurane (DS Pharma Animal Health, Japan) during infection procedures and monitored hourly. Animals that reached predefined humane endpoints were euthanized by sevoflurane overdose (Nikko Pharmaceutical Co., Ltd., Japan) under deep anesthesia, in accordance with AVMA guidelines.

### 2.11 Serum biomarker measurements

At 10 h post-infection, mice were deeply anesthetized and euthanized, and whole-blood samples were collected by cardiac puncture. Blood samples were centrifuged at 1,200 × *g* for 30 min to obtain serum. Serum lactate dehydrogenase (LDH), creatine phosphokinase (CPK), and aspartate aminotransferase (AST) concentrations were measured using a Dimension EXL with the LM Integrated Chemistry System (Siemens, Tokyo, Japan).

### 2.12 Histological examination

At 10 h post-infection, infected thighs were harvested, decalcified, and embedded in paraffin. Sections (4 µm thick) containing both subcutaneous and muscle tissues were prepared and stained with hematoxylin and eosin (H&E). The stained sections were examined and photographed using a light microscope.

### 2.13 Enumeration of bacterial burdens in muscle and in spleen

At 12 h after subcutaneous infection with *A. hydrophila*, infected thigh tissues and spleens were harvested. Muscle tissue directly beneath the inoculation site was dissected, homogenized in 1 mL of 0.1% gelatin in PBS for 5 s using an IKA EUROSTAR homogenizer (IKA Werke, Germany) at 1,300 rpm, and centrifuged at 800 rpm for 5 min. Supernatants were serially diluted 10-fold and plated in duplicate on LB agar plates. After incubation at 37 °C for 24 h, colony-forming units (CFU) were enumerated. Bacterial burdens in spleens were determined in the same manner.

### 2.14 *In vivo* bioluminescence imaging

For *in vivo* bioluminescence imaging, BALB/c mice were used. Bioluminescent WT and mutant strains were visualized using an IVIS-200 imaging system (PerkinElmer, Waltham, MA, USA). Mice were anesthetized with isoflurane, and images were acquired with a fixed exposure time of 1 min at 3, 6, 9, and 12 h after subcutaneous infection. The imaging angle was modified from our previous *V. vulnificus* protocol to better capture bacterial spread within soft tissues (Yamazaki et al., 2022). Imaging experiments were performed using three independent mice per strain.

### 2.15 Statistical analysis

All statistical analyses were performed using GraphPad Prism version 8 (GraphPad Software, San Diego, CA, USA). Comparisons between two groups, including comparisons between each mutant strain and its corresponding complemented strain, were analyzed using the Mann-Whitney U test. Comparisons among more than two groups were analyzed using the Holm-Sidak multiple-comparisons test, with each group compared with WT. Survival curves were compared using the log-rank (Mantel-Cox) test. Data are presented as means ± standard error of the mean (SEM). A *P* value of < 0.05 was considered statistically significant.

### 2.16 Ethics approval

All animal experiments were approved by the Institutional Animal Care and Use Committee of Kitasato University and were performed in accordance with the guidelines of the Japanese Association for Laboratory Animal Science (JALAS) (approval no. 24-033). This study is reported in accordance with the ARRIVE guidelines.

## 3 Results

### 3.1 T2SS-related mutants show reduced colony opacity and attenuated necrosis

**Figure 1:**
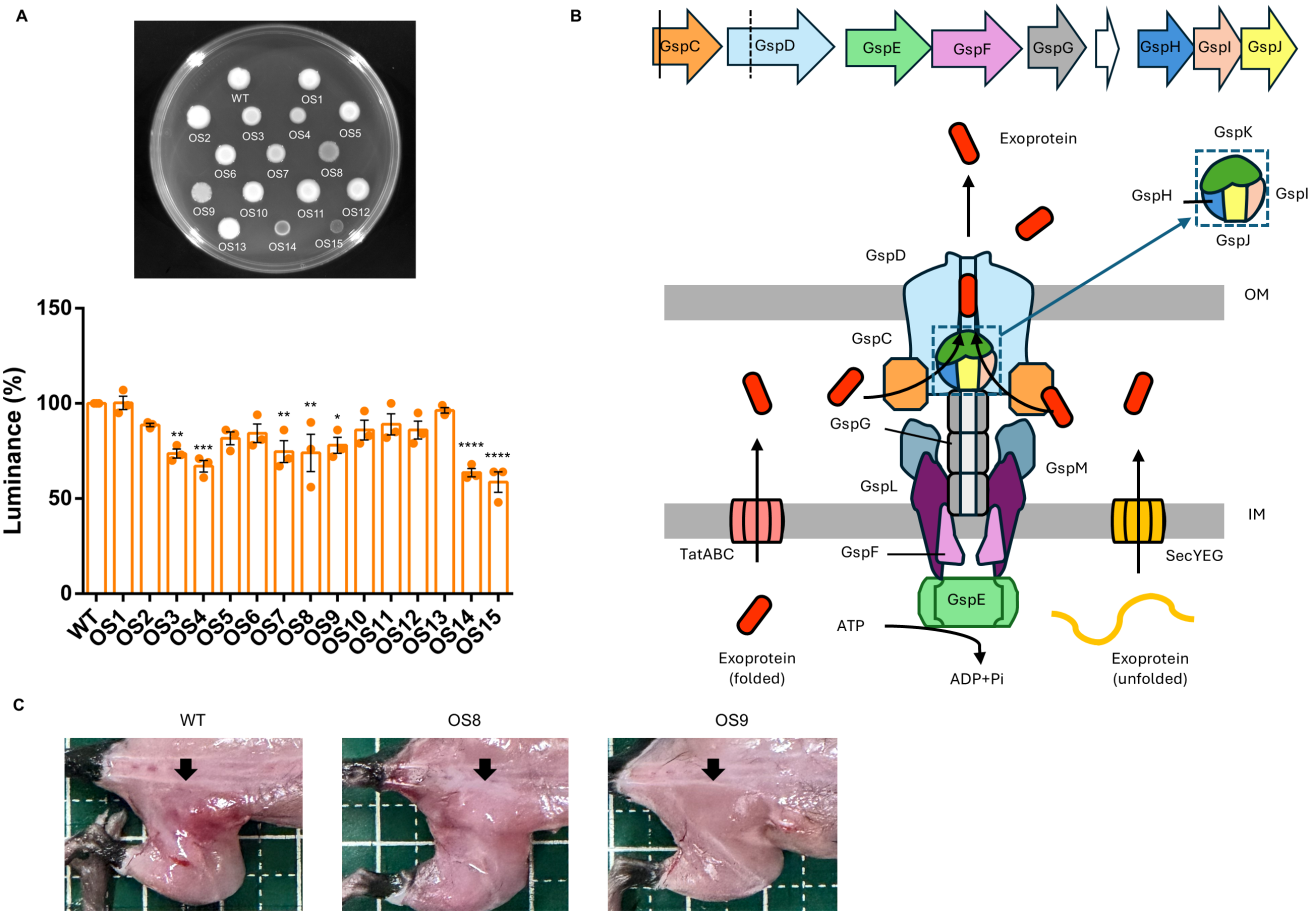
Phenotypic screening of transposon mutants. **(A)** Representative images of colony morphology of the wild-type (WT) strain and transposon mutants (OS1-OS15) grown on LB agar plates. Quantification of colony opacity is shown as relative values normalized to WT (%). Data represent mean ± SEM (n = 3). The image is representative of three independent experiments. Statistical analysis was performed using the Holm-Sidak multiple-comparisons test, with each group compared with WT. *P < 0.05; **P < 0.01; ***P < 0.001; ****P < 0.0001. **(B)** Schematic representation of transposon insertion sites in selected mutants and the organization of the type II secretion system (T2SS). Insertions in *gspC* and *gspD* are indicated. **(C)** *In vivo* screening in a murine NSTI model. Representative gross images of the infection site following subcutaneous inoculation into the right thigh with WT, OS8, or OS9. Arrows indicate the inoculation site.

During phenotypic screening of an *A. hydrophila* transposon insertion mutant library, we identified mutants exhibiting altered colony opacity compared with the wild-type (WT) strain. Because secretion of extracellular virulence factors can influence colony morphology and opacity, colony opacity was used as an initial screening phenotype to identify mutants potentially defective in virulence-associated functions. Bacterial suspensions were spotted onto LB agar plates, and colonies formed after 12 h of incubation were evaluated based on opacity, with translucent variants selected for further analysis. While WT colonies appeared opaque, the Tn mutants OS3, OS4, OS7, OS8, OS9, OS14, and OS15 exhibited reduced colony opacity (**Figure 1A**). To identify the disrupted genes in these mutants, transposon insertion sites were determined (**Table 1**). This analysis revealed that OS8 harbored a transposon insertion in *gspD*, whereas OS9 carried an insertion in *gspC*. Accordingly, OS8 and OS9 were designated the *gspD* mutant and the *gspC* mutant, respectively, in the following analyses (**Figure 1B**). In a mouse NSTI model, WT-infected mice developed severe soft tissue necrosis at the infection site, whereas OS8-infected mice showed only mild necrosis and OS9-infected mice showed little to no necrosis (**Figure 1C**). These results indicate that the GspCD-dependent T2SS contributes to *A. hydrophila* soft tissue pathogenesis.

**Table 1:** Genes disrupted by transposon insertion in screened mutants.

Tn mutant	Disrupted gene/predicted product
OS1	Acetolactate synthase catabolic (EC2.2.1.6)
OS2	Serine hydroxymethyltransferase (EC2.1.2.1)
OS3	Thioredoxin
OS4	Tyrosine-protein kinase Etk/Wzc (EC2.7.10.2)
OS5	Transcription termination factor Rho
OS6	Phage protein
OS7	Phospholipid ABC transporter permease protein MlaE
OS8	General secretion pathway protein D
OS9	General secretion pathway protein C
OS10	Permease of the drug/metabolite transporter (DMT) superfamily
OS11	Hypothetical protein
OS12	Flagellar synthesis regulator FleN
OS13	Glutamine synthetase type I (EC6.3.1.2)
OS14	Dihydrolipoamide dehydrogenase of pyruvate dehydrogenase complex (EC1.8.1.4)
OS15	tRNA-modifying protein YgfZ

### 3.2 Disruption of *gspC* impairs extracellular protein secretion

**Figure 2:**
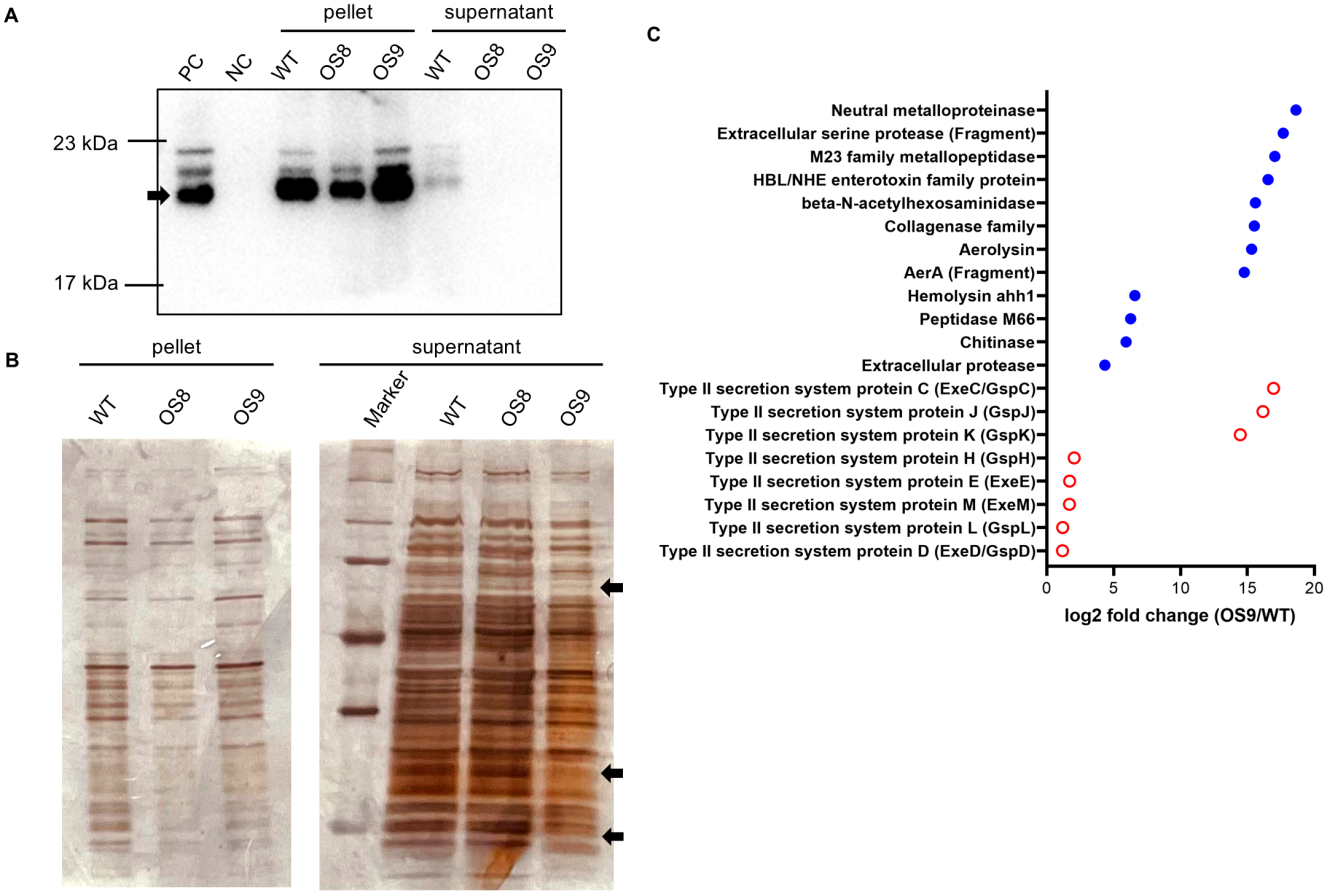
Analysis of extracellular protein secretion. **(A)** Western blot analysis of FLAG-tagged AerA in culture supernatant and pellet fractions from WT, OS8, and OS9. PC, *E. coli* DH5α carrying the Aerolysin expression plasmid; NC, WT *A. hydrophila* without the plasmid. Arrows indicate the FLAG-tagged truncated AerA band. **(B)** Silver staining of proteins in culture supernatant and pellet fractions from WT, OS8, and OS9. **(C)** Representative proteins with reduced abundance in the culture supernatant of the *gspC* mutant (OS9) relative to WT. Proteins are plotted by log2 fold change (OS9/WT). Filled blue circles indicate representative virulence-associated extracellular proteins, and open red circles indicate T2SS-related proteins.

Given that GspC and GspD are essential components of the type II secretion system (T2SS), we hypothesized that T2SS-dependent secretion would be impaired in OS8 and OS9. To assess T2SS function, a FLAG-tagged truncated AerA construct corresponding to the secretion signal and N-terminal region of AerA (predicted molecular mass, approximately 20 kDa) was expressed. FLAG-reactive signals were detected in the culture supernatant of WT, whereas they were markedly reduced in the supernatants of OS8 and OS9 (**Figure 2A**), indicating defective secretion of the truncated AerA construct in these mutants.

To further examine extracellular protein profiles, bacterial cultures were separated into supernatant and pellet fractions and analyzed by silver staining. No notable differences were observed between WT and OS8 in either fraction, whereas three bands present in the WT and OS8 supernatants were markedly reduced in the OS9 supernatant (**Figure 2B**), suggesting a greater alteration in extracellular protein composition in the *gspC* mutant.

Proteomic analysis of culture supernatants from WT and OS9 was then performed to further define these differences. Although the total protein amounts were comparable (**Supplementary Figure 1**), 119 proteins were reduced in OS9 compared with WT (**Supplementary Table 2**). Representative proteins reduced in OS9 included several known or predicted virulence-associated extracellular factors, such as AerA, Ahh, lipase, metalloprotease, collagenase-related proteins, and extracellular serine protease, as well as multiple T2SS-related proteins (**Figure 2C**). Together, these results indicate that disruption of *gspC* broadly impairs extracellular protein secretion in *A. hydrophila*.

### 3.3 T2SS-dependent secretion contributes to hemolytic activity

**Figure 3:**
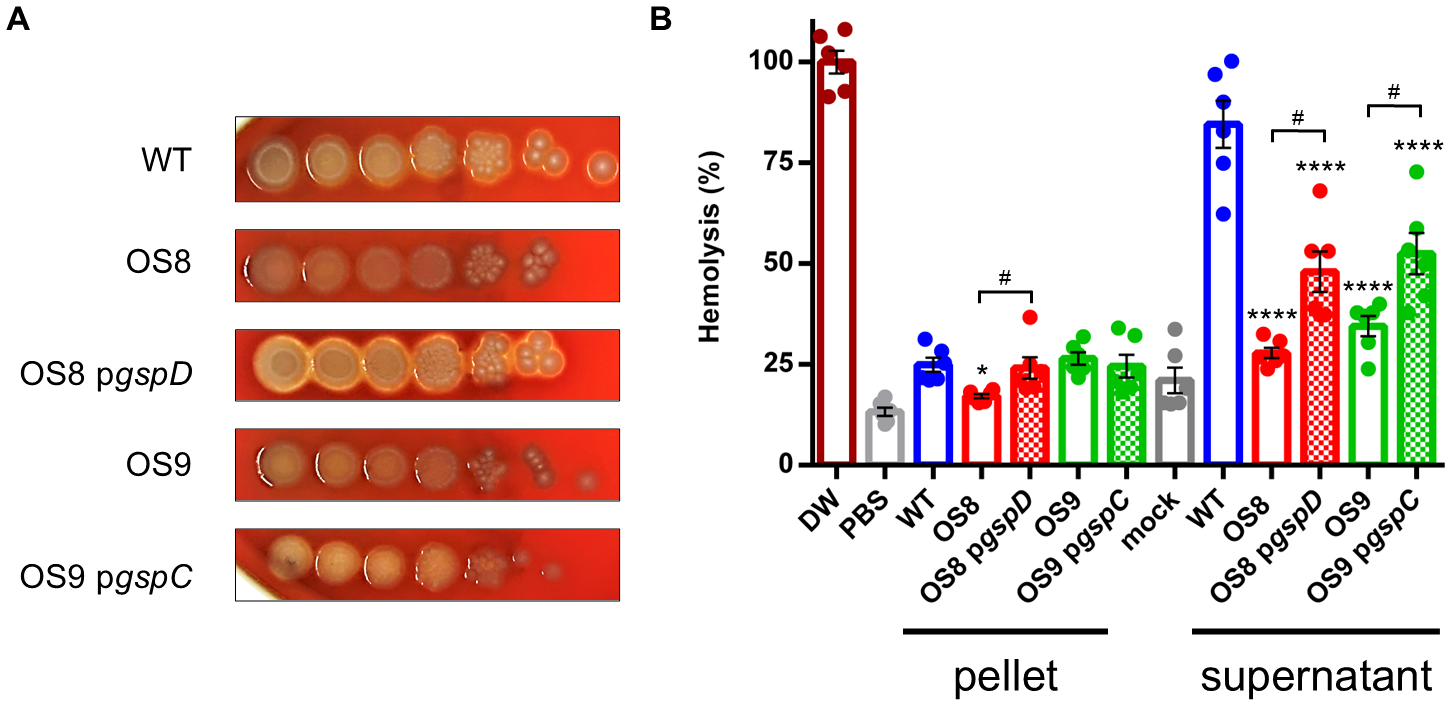
Hemolytic activity of T2SS mutants. **(A)** Hemolysis on blood agar plates by the WT, OS8, OS9 and complemented strains. Representative images from three independent experiments are shown. **(B)** Quantification of hemolytic activity using mouse erythrocytes. Culture supernatant and pellet fractions from each strain were incubated with erythrocytes, and hemolysis was measured by absorbance at 540 nm. Data represent mean ± SEM (n = 6). Statistical analysis was performed using the Holm-Sidak multiple-comparisons test, with each group compared with WT. Comparisons between each mutant and its corresponding complemented strain were performed using the Mann-Whitney U test. Asterisks indicate comparisons with WT, and hash symbols indicate comparisons between each mutant and its corresponding complemented strain. *P < 0.05; ****P < 0.0001; #*P* < 0.01.

We next asked whether disruption of the T2SS affects hemolytic activity. Hemolytic zones were first assessed on blood agar plates. WT formed clear hemolytic zones, whereas no hemolytic zones were observed for OS8 or OS9 (**Figure 3A**). Hemolytic activity was restored in the complemented strains of both mutants.

We further quantified hemolytic activity using mouse erythrocytes. No significant differences were observed among strains in the pellet fraction; however, hemolytic activity in the culture supernatants of OS8 and OS9 was significantly reduced compared with WT, and this reduction was restored in the complemented strains (**Figure 3B**). These findings indicate that T2SS-dependent extracellular factors contribute to hemolytic activity in *A. hydrophila* and support the conclusion that disruption of *gspD* or *gspC* causes the hemolysis-defective phenotype.

### 3.4 T2SS-dependent secretion contributes to soft tissue damage *in vivo*

**Figure 4:**
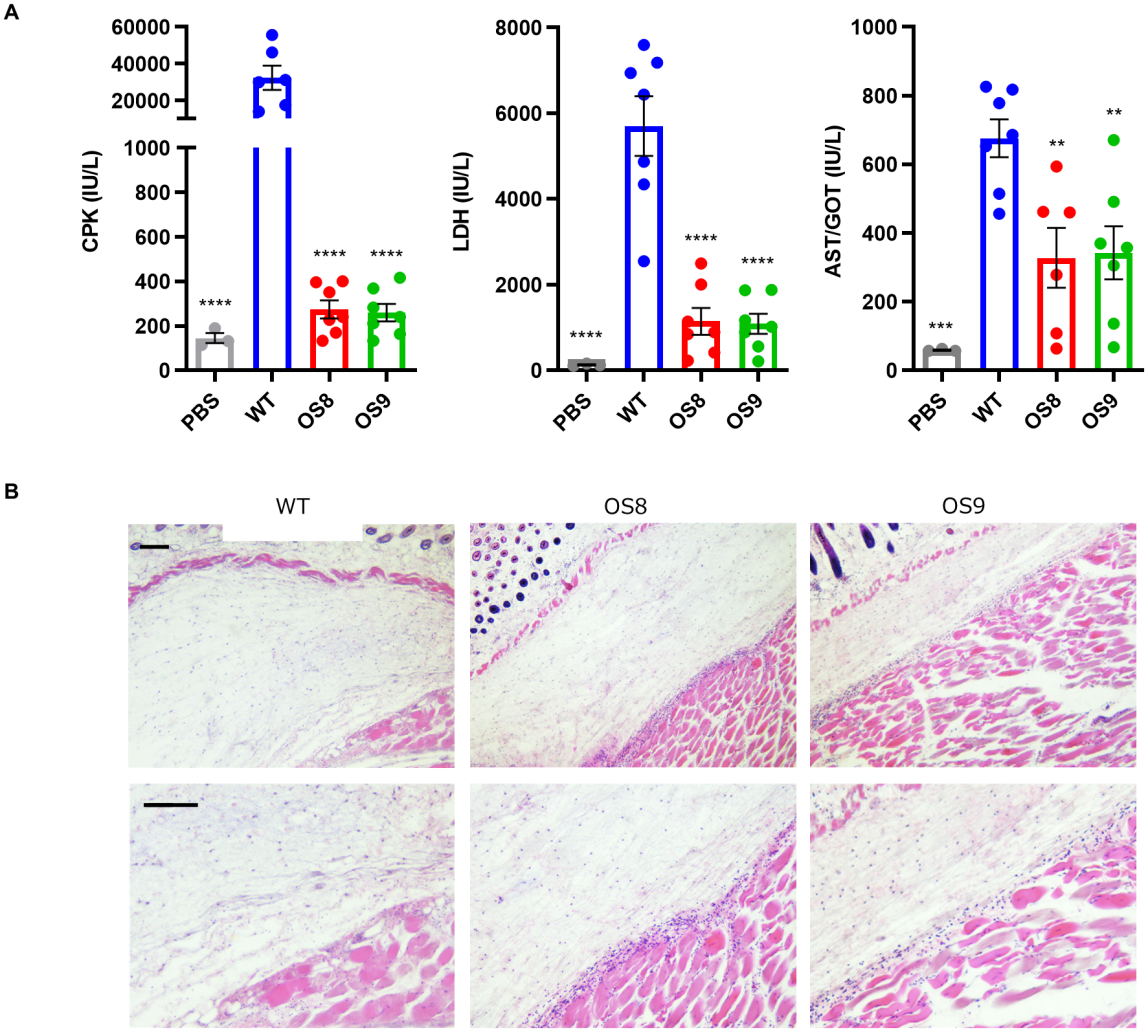
Soft tissue damage in a murine NSTI model. **(A)** Serum levels of CPK, LDH, and AST in mice infected with WT, OS8, or OS9. Data represent mean ± SEM (n = 6). Statistical analysis was performed using the Holm-Sidak multiple-comparisons test, with each group compared with WT. **P < 0.01; ***P < 0.001; ****P < 0.0001. **(B)** Histopathological analysis of infection sites (thigh muscle) from mice infected with WT, OS8, or OS9. Representative H&E-stained sections are shown. The upper panels show low-magnification views, and the lower panels show higher-magnification views of the same sections. All scale bars (100 µm) are shown in the leftmost panels.

We next examined the contribution of T2SS-dependent secretion to tissue damage in the NSTI model. Serum levels of CPK, LDH, and AST were measured in infected mice. All three markers were elevated in WT-infected mice compared with non-infected controls, whereas mice infected with OS8 or OS9 showed lower values than WT-infected mice (**Figure 4A**).

Histopathological analysis of infected tissues at 10 h post-infection revealed marked neutrophil infiltration and degeneration and destruction of muscle cells in WT-infected mice (**Figure 4B**). In contrast, OS8-infected mice showed only mild neutrophil infiltration and cellular damage, whereas OS9-infected mice exhibited minimal pathological changes (**Figure 4B**). These histopathological findings were consistent with the gross pathological changes observed at the infection site (**Figure 1C**). Collectively, these results indicate that T2SS-dependent extracellular factors contribute substantially to soft tissue damage during *A. hydrophila* infection.

### 3.5 T2SS-dependent secretion promotes *in vivo* expansion and dissemination

**Figure 5:**
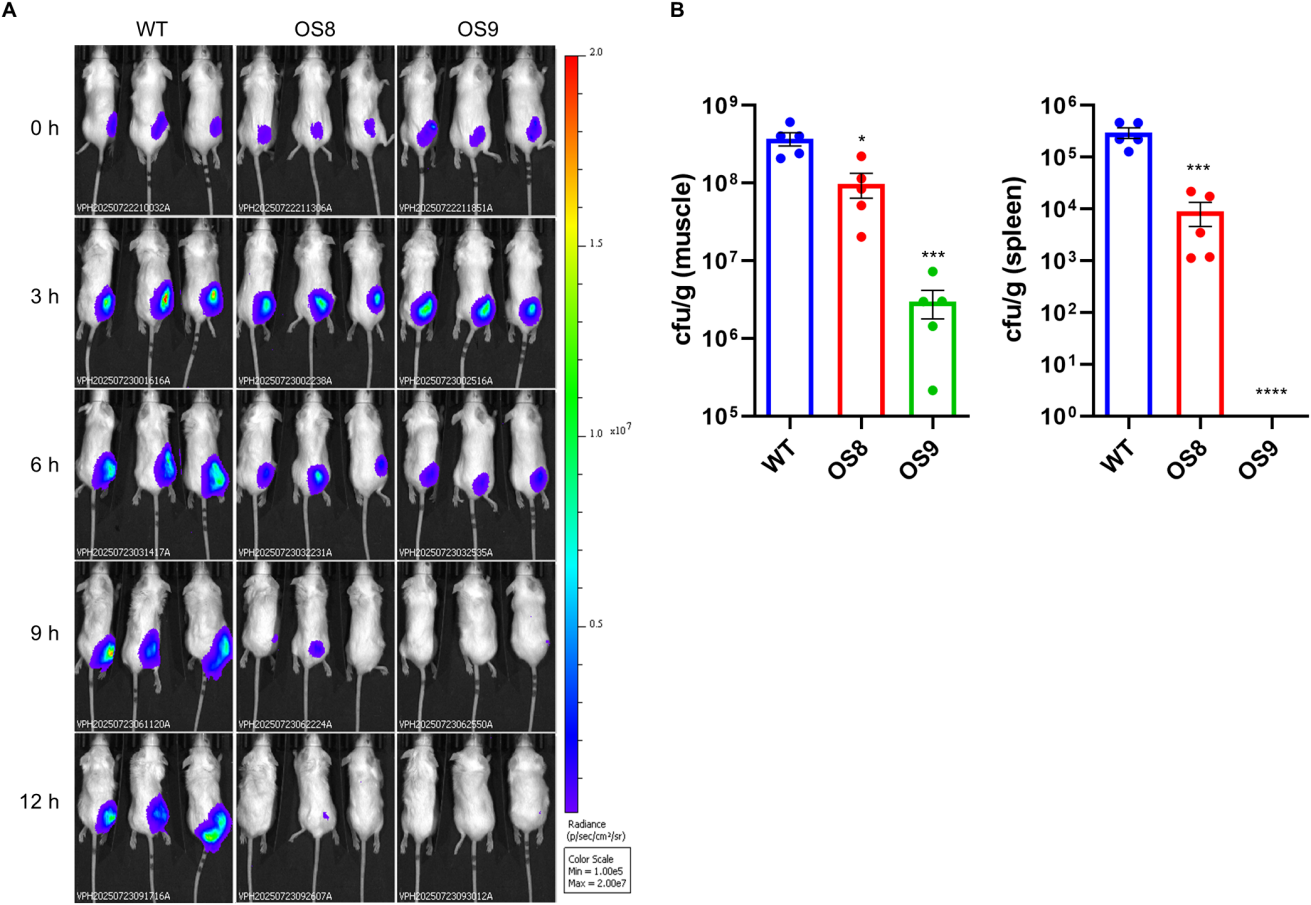
*In vivo* bacterial proliferation and dissemination. **(A)** Bioluminescent signals of WT, OS8, and OS9 were monitored at the indicated time points following subcutaneous infection in a murine NSTI model. Images were acquired using identical imaging parameters. Representative bioluminescence images are shown (n = 3 per group). **(B)** Bacterial burdens in muscle at the infection site and in the spleen of mice infected with WT, OS8, or OS9. Data represent mean ± SEM (n = 6). Statistical analysis was performed using the Holm-Sidak multiple-comparisons test, with each group compared with WT. *P < 0.05; ***P < 0.001; ****P < 0.0001.

To evaluate the impact of T2SS-dependent tissue damage on bacterial behavior *in vivo*, bioluminescent *A. hydrophila* strains carrying chromosomally integrated *luxCDABE* were used to monitor bacterial expansion and dissemination by IVIS analysis. In WT-infected mice, signal intensity increased after 6 h post-infection and spread beyond the inoculation site. In contrast, signal intensity decreased after 6 h in OS8- and OS9-infected mice, with signals nearly disappearing by 12 h post-infection (**Figure 5A**).

To assess local expansion and systemic dissemination, bacterial burdens were quantified in muscle tissue and in the spleen. WT-infected mice showed higher bacterial burdens in both tissues than mice infected with OS8 or OS9 (**Figure 5B**). Moreover, OS9 was more strongly attenuated than OS8, and no bacteria were detected in the spleens of OS9-infected mice (**Figure 5B**). These results indicate that T2SS-dependent secretion promotes bacterial expansion at the infection site and dissemination to systemic organs *in vivo*.

### 3.6 T2SS-dependent secretion is critical for host lethality

**Figure 6:**
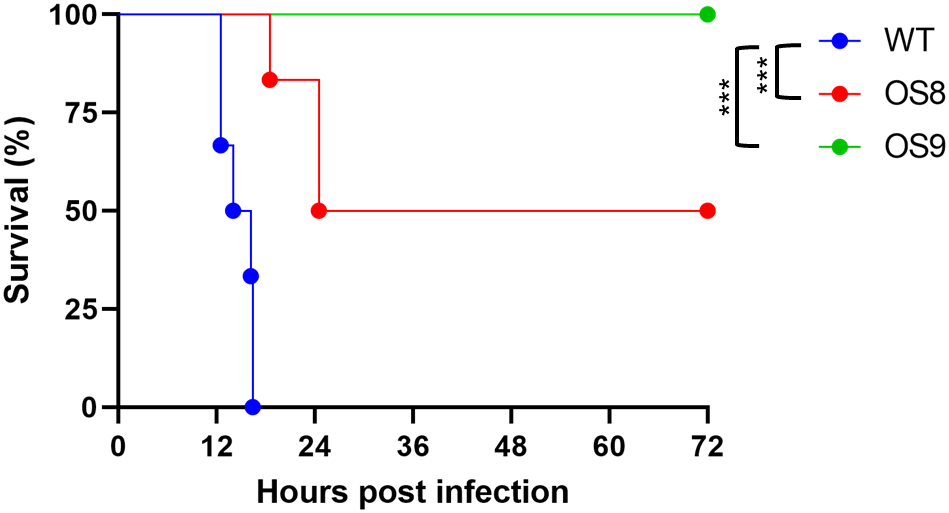
Survival of mice infected with T2SS mutants. Survival curves of mice infected with WT, OS8, or OS9. Mice were monitored over the indicated period following subcutaneous infection in a murine NSTI model (n = 6 per group). Statistical analysis was performed using the log-rank (Mantel–Cox) test. ***P < 0.001.

Finally, we assessed the contribution of T2SS-dependent secretion to host lethality using the mouse NSTI model. All mice infected with WT died within 20 h post-infection (**Figure 6**). In contrast, some mice infected with OS8 survived throughout the observation period, whereas OS9 caused no deaths (**Figure 6**). These results show that T2SS-dependent secretion is critical for the lethal outcome of *A. hydrophila* soft tissue infection.

## 4 Discussion

*Aeromonas hydrophila* causes necrotizing soft tissue infections (NSTIs), a severe disease characterized by rapid progression and high mortality (Devos et al., 2020; Gold and Salit, 1993). In this study, we identified the GspCD-dependent type II secretion system (T2SS) as an important virulence mechanism of *A. hydrophila* in NSTIs. Disruption of *gspC* or *gspD* impaired secretion of a FLAG-tagged truncated AerA construct, reduced hemolytic activity, attenuated tissue damage, decreased *in vivo* expansion and dissemination, and markedly reduced lethality. These findings indicate that the T2SS contributes not only to extracellular protein secretion but also to the progression of invasive and lethal soft tissue infection.

The initial screen was based on colony opacity, which likely reflects changes in extracellular structures or secreted products (**Figure 1A**). Using this phenotype, we identified mutants with transposon insertions in *gspD* and *gspC*. Both OS8 and OS9 were impaired in T2SS-dependent secretion, as demonstrated by the loss of FLAG-tagged AerA signals in culture supernatants (**Figure 2A**). Because Aerolysin and hemolysins are membrane-active pore-forming toxins (Fernandez-Bravo and Figueras, 2020; Abrami et al., 2000; Galindo et al., 2006), the reduced hemolytic activity observed in both mutants is consistent with impaired secretion of these factors.

In addition, proteomic analysis revealed reduced abundance of multiple known or predicted virulence-associated extracellular proteins in the supernatant of the *gspC* mutant, including AerA, Ahh, lipase, metalloprotease, collagenase-related proteins, extracellular serine protease, and several T2SS-related proteins (**Figure 2C** and **Supplementary Table 2**). These findings indicate that disruption of *gspC* substantially alters the extracellular protein profile associated with *A. hydrophila* virulence.

Although whole-genome sequencing confirmed the absence of additional transposon insertions in OS8 and OS9, this study utilized transposon insertion mutants rather than clean allelic exchange mutants, and subtle polar effects cannot be completely excluded. Restoration of hemolysis by complementation further supports the requirement of *gspD* and *gspC* for this phenotype (**Figures 3A, B**). Proteomic analyses were not performed using the complemented strains. Therefore, additional studies will be required to fully define the contribution of individual T2SS-dependent proteins to the phenotypes observed in this study.

In our recent study, transposon insertion mutants were also used for phenotypic characterization, and plasmid-based complementation restored the relevant phenotypes more clearly (Yamazaki et al., 2026a). In contrast, the disrupted genes examined here encode structural components of the T2SS. Because the T2SS is a multicomponent secretion apparatus, plasmid-based complementation of a single component may not be sufficient to fully restore native assembly, stoichiometry, or regulation during infection. This structural and regulatory complexity may account for the incomplete complementation observed in the present study.

The *in vivo* data directly link these secretion defects to disease pathogenesis. In the NSTI model, WT infection caused marked increases in serum CPK, LDH, and AST, together with pronounced neutrophil infiltration and severe muscle damage (**Figures 4A, B**). These changes were attenuated in the *gspD* mutant and were minimal in the *gspC* mutant, consistent with the gross pathological changes observed at the infection site. Although bioluminescent signals of OS8 and OS9 increased during the first 3 h after infection, both mutants subsequently showed reduced signal intensity compared with WT (**Figure 5A**). This pattern suggests that disruption of the T2SS does not markedly impair bacterial growth during the initial phase of infection. The subsequent decline in signal intensity may reflect an increased requirement for T2SS-dependent factors during later stages of infection, thereby limiting the persistence and/or expansion of the mutants. A similar temporal pattern has also been observed in our recent *Vibrio vulnificus* NSTI study, in which certain mutants showed early signal establishment followed by impaired persistence or dissemination in host tissues (Yamazaki et al., 2026a). In addition, WT showed robust expansion at the infection site and dissemination to the spleen, whereas both mutants were markedly impaired in these processes, and no bacteria were detected in the spleens of mice infected with OS9 (**Figure 5B**). Together with the IVIS results, these findings indicate that T2SS-dependent secretion contributes not only to host tissue injury but also to bacterial expansion and dissemination *in vivo*. Furthermore, complementation improved hemolytic activity *in vitro*, although the phenotype was not fully restored to the WT level (**Figure 3B**). *In vivo* lethality was not restored in either the complemented *gspD* strain or the complemented *gspC* strain under the conditions tested (**Supplementary Figure 2**). These findings support the interpretation that disruption of *gspC* and *gspD* contributed substantially to the attenuated phenotypes observed in OS8 and OS9, while also indicating that plasmid-based complementation did not fully restore T2SS function *in vivo*.

We previously showed that motility is required for efficient spread of *A. hydrophila* in soft tissues (Yamazaki et al., 2021). The present study extends this model by demonstrating that, in addition to motility, T2SS-dependent extracellular factors are required for efficient tissue destruction, local expansion, systemic spread, and lethal outcome during NSTIs. These observations support a model in which bacterial motility and T2SS-dependent secretion act cooperatively during soft tissue infection, with motility promoting spread through tissue and secreted factors facilitating destruction of host barriers and progression of necrotic disease.

In many Gram-negative bacteria, GspD is considered the key structural component of the T2SS because it forms the outer membrane secretion channel (Burtnick et al., 2014; Chowdhury and Heinemann, 2006; Douzi et al., 2012; Sandkvist et al., 1997; Tipton et al., 2015; Filloux, 2004), whereas GspC is generally thought to function in substrate recognition and linkage of the secretion machinery (Parker et al., 1996; Korotkov et al., 2012; Sandkvist et al., 1997). Although differences in the severity of attenuation were observed between the *gspC* and *gspD* mutants across several assays, the present study was not designed to determine the relative contribution of individual T2SS components to virulence. Rather, our findings demonstrate that disruption of either component impairs T2SS-dependent secretion and pathogenicity. Further studies will be required to clarify whether individual T2SS components contribute differently to secretion efficiency or virulence in *A. hydrophila*. In summary, our findings establish the GspCD-dependent T2SS as an important contributor to *A. hydrophila* NSTI pathogenesis. Rather than acting through a single toxin alone, this secretion system promotes the extracellular export of multiple factors associated with hemolysis, tissue destruction, *in vivo* expansion, dissemination, and host mortality. These results highlight the importance of T2SS-dependent secretion in severe soft tissue infection and provide new insight into the molecular pathogenesis of *A. hydrophila* NSTIs.

## Data Availability

The datasets generated and analyzed during the current study are openly available in Zenodo at https://doi.org/10.5281/zenodo.20593816 (Tonosaki et al., 2026).
