# Supplementary figures and images for: The GspCD-dependent type II secretion system promotes necrotizing soft tissue infection caused by Aeromonas hydrophila
Source: Front Cell Infect Microbiol. 2026 Jun 30;16:1870837. doi: 10.3389/fcimb.2026.1870837 (PMC13364864; doi:10.3389/fcimb.2026.1870837)

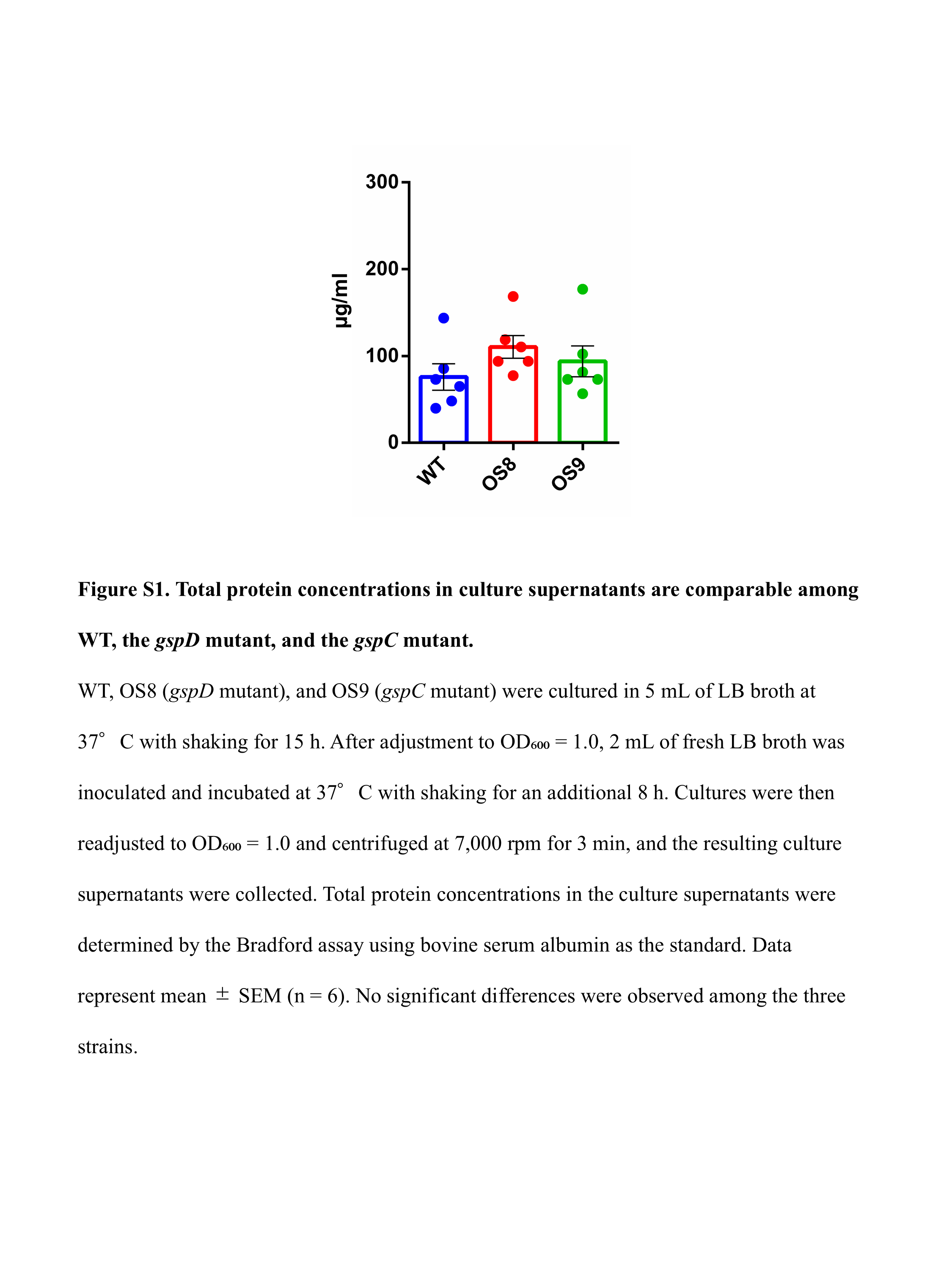

Supplement: Supplementary file 1 [file Image1.tif]

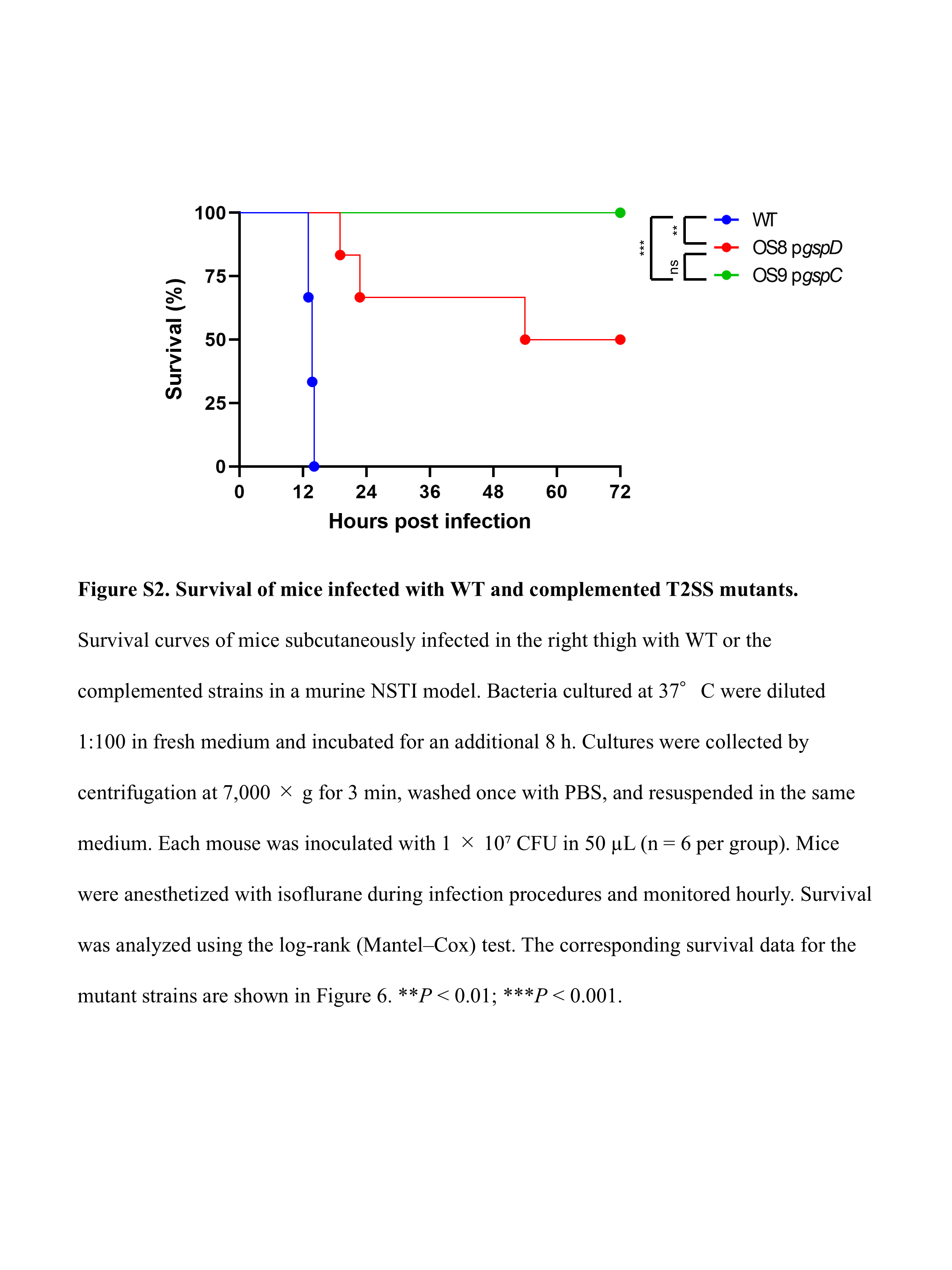

Supplement: Supplementary file 2 [file Image2.tif]
