# Supplementary material for: The GspCD-dependent type II secretion system promotes necrotizing soft tissue infection caused by Aeromonas hydrophila
Source: Front Cell Infect Microbiol. 2026 Jun 30;16:1870837. doi: 10.3389/fcimb.2026.1870837 (PMC13364864; doi:10.3389/fcimb.2026.1870837)
Supplement: Supplementary file 3 [file Table1.docx]

Table S1. Arbitrarily primed PCR primers

| **Primer ID** | **Primer name** | **PCR step / orientation** | **Sequence (5′→3′)** |
| --- | --- | --- | --- |
| P132 | Arb1 | 1st AP-PCR | ggccacgcgtcgactagtcannnnnnnnnngatat |
| P162 | Arb3 | 1st AP-PCR | ggccacgcgtcgactagtcannnnnnnnnnttcaa |
| P163 | Arb4 | 1st AP-PCR | ggccacgcgtcgactagtcannnnnnnnnnccacg |
| P164 | Arb5 | 1st AP-PCR | ggccacgcgtcgactagtcannnnnnnnnnactga |
| P133 | Arb6 | 1st AP-PCR | ggccacgcgtcgactagtcannnnnnnnnnacgcg |
| P165 | Arb7 | 1st AP-PCR | ggccacgcgtcgactagtcannnnnnnnnntggca |
| P488 | Arb8 | 1st AP-PCR | ggccacgcgtcgactagtcannnnnnnnnnatgcca |
| P489 | Arb9 | 1st AP-PCR | ggccacgcgtcgactagtcannnnnnnnnncgggct |
| P490 | Arb10 | 1st AP-PCR | ggccacgcgtcgactagtcannnnnnnnnncaatat |
| p144 | consensus1 I-out 1st | 1st AP-PCR, forward insertion orientation | ggtacctacaacctcaagct |
| p166 | Rev consensus2 I-out 1st | 1st AP-PCR, reverse insertion orientation | ccatgggtaagattggttcgaa |
| P134 | Arb2nd | 2nd AP-PCR | ggccacgcgtcgactagtca |
| p145 | consensus2 I-out 2nd | 2nd AP-PCR, forward insertion orientation | gcttggttagaatgggtacc |
| p167 | Rev consensus1 I-out 2nd | 2nd AP-PCR, reverse insertion orientation | agcttggttagaatgggtacc |
