# Supplementary material for: The GspCD-dependent type II secretion system promotes necrotizing soft tissue infection caused by Aeromonas hydrophila
Source: Front Cell Infect Microbiol. 2026 Jun 30;16:1870837. doi: 10.3389/fcimb.2026.1870837 (PMC13364864; doi:10.3389/fcimb.2026.1870837)
Supplement: Supplementary file 4 [file Table2.docx]

Table S2. Proteins with reduced abundance in the culture supernatant of the *gspC* mutant (OS9) relative to WT

| Gene | Protein description | WT | OS9 | Fold change (OS9/WT) | log2FC (OS9/WT) |
| --- | --- | --- | --- | --- | --- |
| aerA | AerA (Fragment) | 27721.4 | 1 | 3.60732E-05 | 14.758713 |
| aerA | Aerolysin | 40701.3 | 1 | 2.45692E-05 | 15.312787 |
|  | Aerolysin (Fragment) | 57033.2 | 1 | 1.75336E-05 | 15.799514 |
| C1A23_02595 | Amino acid ABC transporter substrate-binding protein | 144056 | 1 | 6.94174E-06 | 17.13627 |
| AHA_1528;C1A23_10305;JAJ28_001713 | beta-N-acetylhexosaminidase | 49435.6 | 1 | 2.02283E-05 | 15.593263 |
| AHA_2650;C1A23_16315;JAJ28_002680;PY771_12010 | Cation transporter | 85146.7 | 1 | 1.17444E-05 | 16.377663 |
| AHA_0517 | Collagenase family | 46664.6 | 1 | 2.14295E-05 | 15.510041 |
| AHA_3705;C1A23_04920;pbpG | D-alanyl-D-alanine endopeptidase | 71463.9 | 1 | 1.39931E-05 | 16.124927 |
| AHA_3948;C1A23_03700;JAJ28_002962 | DUF3103 domain-containing protein | 1083850 | 1 | 9.22637E-07 | 20.047734 |
| AHA_1223;C1A23_08545;JAJ28_003911 | DUF882 domain-containing protein | 77989.8 | 1 | 1.28222E-05 | 16.250998 |
| AHA_3402;C1A23_20500;PY771_16190 | Endonuclease | 54442.3 | 1 | 1.83681E-05 | 15.73244 |
| ahpA | Extracellular serine protease (Fragment) | 208469 | 1 | 4.79688E-06 | 17.669473 |
| AHA_2582;C1A23_15970;JAJ28_004123 | HBL/NHE enterotoxin family protein | 95312.9 | 1 | 1.04918E-05 | 16.540384 |
| JAJ28_002869;PY771_08215 | Insulinase family protein | 54080.6 | 1 | 1.84909E-05 | 15.722824 |
| AHA_3401;C1A23_20495;JAJ28_001330 | Lipoprotein | 74572.9 | 1 | 1.34097E-05 | 16.186364 |
| C1A23_08935;PY771_03710 | M23 family metallopeptidase | 135279 | 1 | 7.39213E-06 | 17.045578 |
| C1A23_12080;JAJ28_003159;PY771_11255 | NADH-quinone oxidoreductase subunit B family protein | 74895.4 | 1 | 1.3352E-05 | 16.192589 |
| AHA_0851;C1A23_06470;JAJ28_003496;PY771_17080 | Neutral metalloproteinase | 405250 | 1 | 2.46761E-06 | 18.628453 |
| prfC | Peptide chain release factor 3 | 50091.4 | 1 | 1.99635E-05 | 15.612275 |
| exeC | Type II secretion system protein C | 125920 | 1 | 7.94155E-06 | 16.942148 |
| exeJ;gspJ | Type II secretion system protein J | 73199.8 | 1 | 1.36612E-05 | 16.159552 |
| C1A23_22405;exeK;gspK | Type II secretion system protein K | 22644.7 | 1 | 4.41604E-05 | 14.466886 |
| C1A23_19345;PY771_14960 | Uncharacterized protein | 75351.6 | 1 | 1.32711E-05 | 16.201351 |
| aerA;hly;pznHA | Hemolysin (Fragment) | 17217900 | 122741 | 0.007128686 | 7.1321481 |
| ahh1 | Hemolysin ahh1 | 26475400 | 276377 | 0.010439011 | 6.5818711 |
| JAJ28_000746 | Peptidase M66 | 12909300 | 166397 | 0.0128897 | 6.2776375 |
| aerA | Beta-barrel pore-forming toxin aerolysin | 9487820 | 142936 | 0.01506521 | 6.0526354 |
|  | Haemolysin (Fragment) | 30566000 | 463770 | 0.015172741 | 6.0423745 |
| JAJ28_000747 | Chitinase | 7099490 | 116567 | 0.016419067 | 5.9284841 |
| PY771_23435 | DUF3103 family protein | 12104700 | 201151 | 0.016617595 | 5.9111446 |
| AHA_1304;C1A23_08930;JAJ28_003966;PY771_03705 | Alpha-amylase | 753067 | 12652 | 0.01680063 | 5.8953409 |
| C1A23_07130 | Peptidase M66 | 3491710 | 61172.7 | 0.01751941 | 5.834902 |
| AHA_0610 | Lytic polysaccharide monooxygenase | 4157230 | 87071.7 | 0.020944644 | 5.5772748 |
| JAJ28_000231 | ExeM/NucH family extracellular endonuclease | 420276 | 11546.4 | 0.027473376 | 5.185822 |
| AHA_0977;JAJ28_000745 | chitinase | 5496720 | 152153 | 0.02768069 | 5.1749763 |
| JAJ28_002968 | Chitinase | 1728890 | 54302 | 0.031408592 | 4.9926969 |
| AHA_3791;C1A23_04495;JAJ28_000447;LCAT;PY771_24230 | SGNH/GDSL hydrolase family protein | 9940370 | 349141 | 0.035123542 | 4.8314179 |
| AHA_2713;C1A23_16680;JAJ28_004196;epr;mepA | Peptidase M35 | 2536650 | 91756.7 | 0.036172394 | 4.7889671 |
| ahe2 | Serine protease Ahe2 | 905818 | 33424.1 | 0.036899357 | 4.7602605 |
| PY771_18840 | Collagenase | 4325080 | 159883 | 0.036966484 | 4.7576384 |
| AHA_2687;PY771_12195;serA | S8 family serine peptidase | 2930850 | 108914 | 0.037161233 | 4.7500578 |
| AHA_2363 | Chitinase-1 | 4778750 | 228772 | 0.04787277 | 4.3846509 |
| eprA1 | Extracellular protease | 318757 | 15617.5 | 0.048995002 | 4.3512216 |
| lamB | Maltoporin (Fragment) | 1361510 | 101110 | 0.074263134 | 3.75121 |
|  | Aerolysin-like toxin (Fragment) | 1049200 | 93166 | 0.088797179 | 3.4933423 |
| C1A23_13210 | ExeM/NucH family extracellular endonuclease | 504781 | 45427.2 | 0.089993877 | 3.4740293 |
| lamB | Maltoporin | 556361 | 52977.5 | 0.095221448 | 3.3925696 |
| AHA_2764;C1A23_16935;JAJ28_000526 | OmcA/MtrC family decaheme c-type cytochrome | 127583 | 12910.3 | 0.10119138 | 3.3048417 |
| C1A23_22195 | Peptidase M12B domain-containing protein | 372092 | 39067.4 | 0.104993922 | 3.2516223 |
| lamB | Maltoporin | 7013270 | 777661 | 0.110884224 | 3.172874 |
| AHA_3478;Asp;C1A23_21045;JAJ28_004075;PY771_01475 | S8 family peptidase | 110771 | 16044.8 | 0.144846574 | 2.7874025 |
| JAJ28_001367 | DUF1460 domain-containing protein | 244917 | 38042.3 | 0.155327318 | 2.6866165 |
| PY771_09555 | ExeM/NucH family extracellular endonuclease | 262104 | 51894.5 | 0.197992018 | 2.3364858 |
|  | Alpha-amylase | 222445 | 47243 | 0.212380588 | 2.2352762 |
| PY771_16635 | ExeM/NucH family extracellular endonuclease | 1168770 | 264494 | 0.226301154 | 2.1436842 |
| AHA_3762;C1A23_04640;JAJ28_000418 | Esterase YqiA | 266067 | 60894.2 | 0.228867914 | 2.1274129 |
| pulA | Pullulanase-type alpha-1,6-glucosidase | 86325.3 | 20093 | 0.232759118 | 2.1030904 |
| AHA_3403;C1A23_20505;JAJ28_001332;PY771_16195 | Cytochrome-c peroxidase | 137143 | 32617.2 | 0.237833497 | 2.0719762 |
| exeH;gspH | Type II secretion system protein H | 26406.8 | 6382.46 | 0.241697584 | 2.048725 |
| C1A23_04290 | DUF885 domain-containing protein | 1005000 | 247680 | 0.246447761 | 2.0206462 |
| AHA_3182;C1A23_19390;JAJ28_001013 | Uncharacterized protein | 28848.7 | 7351.81 | 0.254840262 | 1.9723349 |
| AHA_1043 | Peptidase, U32 family | 62924 | 16404.7 | 0.260706554 | 1.9395012 |
| pulA | Pullulanase-type alpha-1,6-glucosidase | 1807500 | 491133 | 0.271719502 | 1.87981 |
| mepA | Penicillin-insensitive murein endopeptidase | 76755.8 | 21041.9 | 0.274140863 | 1.8670107 |
| C1A23_21110 | Retention module-containing protein | 136852 | 41706.4 | 0.304755491 | 1.7142759 |
| exeE | Type II secretion system protein E | 78809.4 | 24108.3 | 0.305906418 | 1.7088377 |
| exeM | Type II secretion system protein M | 74684.8 | 22962.6 | 0.30746016 | 1.7015286 |
| AHA_3265;PY771_15440 | Alpha/beta hydrolase | 11660.1 | 3633.74 | 0.311638846 | 1.682053 |
| AHA_3789;mltC | Membrane-bound lytic murein transglycosylase MltC | 44964 | 14161.7 | 0.314956414 | 1.6667759 |
| C1A23_15965;JAJ28_004124;PY771_11655 | HBL/NHE enterotoxin family protein | 56239.7 | 17896.1 | 0.318211154 | 1.6519437 |
| AHA_3173 | Uncharacterized protein | 74916.3 | 25886.8 | 0.345542984 | 1.5330629 |
| AHA_4190;C1A23_02550;JAJ28_001135;PY771_21300 | DUF5610 domain-containing protein | 347267 | 120235 | 0.34623215 | 1.5301884 |
| AHA_0837;JAJ28_003511 | Alpha-amylase | 225353 | 80069.2 | 0.35530569 | 1.4928673 |
|  | General secretion pathway protein D | 297966 | 108594 | 0.364450978 | 1.4562033 |
| slmA | Nucleoid occlusion factor SlmA | 214034 | 78180.6 | 0.36527188 | 1.4529574 |
| C1A23_19340 | Alpha-glucosidase | 20995.5 | 7945.56 | 0.378441097 | 1.4018593 |
| AHA_2515;hypB | Hydrogenase maturation factor HypB | 61714.3 | 24806.6 | 0.401958692 | 1.3148808 |
| C1A23_06750;JAJ28_003677 | DUF2860 domain-containing protein | 332588 | 134702 | 0.405011606 | 1.3039648 |
| rnfG | Ion-translocating oxidoreductase complex subunit G | 102903 | 42157.1 | 0.409678061 | 1.2874375 |
| fruA;fruA_2 | protein-N(pi)-phosphohistidine--D-fructose phosphotransferase | 76968 | 32298.2 | 0.419631525 | 1.252805 |
| gspL | Type II secretion system protein L | 139880 | 61398 | 0.438933371 | 1.1879261 |
| exeD;gspD | Type II secretion system protein GspD | 88966.4 | 39135 | 0.439885178 | 1.1848011 |
| AHA_3492 | Putative transport protein AHA_3492 | 215042 | 94835.2 | 0.441007818 | 1.1811239 |
| C1A23_22395;exeM | Type II secretion system protein M | 75992.3 | 34932.5 | 0.45968475 | 1.1212833 |
| AHA_3483 | Aminopeptidase Y | 439244 | 202662 | 0.461388203 | 1.115947 |
| JAJ28_000773;PY771_02080 | Tetratricopeptide repeat protein | 72417.2 | 33514 | 0.462790588 | 1.1115686 |
| PY771_08620 | FAD-binding protein | 455432 | 214527 | 0.471040682 | 1.0860764 |
| AHA_3100;C1A23_18960;JAJ28_004284;PY771_14365 | beta-N-acetylhexosaminidase | 28722.9 | 13531.1 | 0.471090991 | 1.0859224 |
| JAJ28_000488 | DUF885 domain-containing protein | 120866 | 57533.9 | 0.47601392 | 1.0709243 |
| AHA_0495;C1A23_22830;PY771_18945 | Glycosidase | 156388 | 74817.9 | 0.478412017 | 1.0636745 |
| AHA_0721;JAJ28_002367;PY771_17800 | DUF4432 family protein | 233651 | 112174 | 0.480092103 | 1.0586169 |
| exeN | Type II secretion system protein N | 69807.8 | 33566.3 | 0.48083885 | 1.0563746 |
| phoB | Phosphate regulon transcriptional regulatory protein PhoB | 199284 | 97222.2 | 0.487857546 | 1.0354682 |
| ppx | Exopolyphosphatase | 70675.8 | 35069.1 | 0.496196762 | 1.0110158 |
| PY771_09120 | Glycoside hydrolase family 1 protein | 705909 | 361153 | 0.511614103 | 0.9668721 |
| PY771_14165 | Formate dehydrogenase subunit alpha | 1093680 | 566977 | 0.51841215 | 0.9478286 |
| AHA_1091;C1A23_07855;JAJ28_000881;PY771_02375 | LPP20 family lipoprotein | 32196.5 | 16729.8 | 0.51961551 | 0.9444836 |
| C1A23_23390 | Prepilin-type N-terminal cleavage/methylation domain-containing protein | 349462 | 182805 | 0.523104086 | 0.9348301 |
| exeL | Type II secretion system protein L | 106891 | 55990.1 | 0.523805574 | 0.9328967 |
| AHA_0149;C1A23_24705;JAJ28_003684;PY771_22850 | Bifunctional acetate--CoA ligase family protein/GNAT family N-acetyltransferase | 23952.3 | 12987.7 | 0.542231843 | 0.8830183 |
| ahlip9 | Lipase | 1565140 | 871640 | 0.556908647 | 0.8444874 |
| C1A23_22800;PY771_18915 | Dipeptidase | 97223.9 | 54214.8 | 0.557628337 | 0.8426242 |
| AHA_1882 | TIGR01620 family protein | 276269 | 155818 | 0.564008267 | 0.8262118 |
| AHA_1342 | Oxidoreductase alpha (Molybdopterin) subunit | 241096 | 138956 | 0.576351329 | 0.7949796 |
| AHA_1792 | Oxidoreductase, aldo/keto reductase family | 230169 | 133187 | 0.578648732 | 0.7892403 |
| JAJ28_000405 | Uncharacterized protein | 77118.5 | 44663.7 | 0.579156742 | 0.7879742 |
| AHA_1305 | Protease LasA | 108651 | 63322.2 | 0.582803649 | 0.7789182 |
| AHA_0165;JAJ28_003702;PY771_22930 | DUF535 domain-containing protein | 151753 | 88591.4 | 0.583786801 | 0.7764865 |
| AHA_1406 | Peptidase M12B domain-containing protein | 87074.7 | 51779.3 | 0.594653773 | 0.7498782 |
| mglC | Galactose/methyl galactoside ABC transporter permease MglC | 90977.7 | 54545.3 | 0.599545811 | 0.7380581 |
| AHA_2524;hybB | Ni/Fe-hydrogenase cytochrome b subunit | 72076.1 | 43626.8 | 0.60528802 | 0.7243063 |
| lip | Extracellular lipase | 1053540 | 639312 | 0.606822712 | 0.720653 |
| AHA_0115;C1A23_24885 | 6-phospho-alpha-glucosidase | 71665.2 | 43536.1 | 0.607492893 | 0.7190606 |
| cysN | Sulfate adenylyltransferase subunit 1 | 108283 | 65785 | 0.607528421 | 0.7189762 |
| gt6 | Putative glycosyltransferase | 143415 | 88583.2 | 0.617670419 | 0.6950909 |
| AHA_2525;hybA | Hydrogenase 2 operon protein HybA | 202989 | 125525 | 0.618383262 | 0.6934268 |
| AHA_1412;C1A23_09525;PY771_04785 | Cupin domain-containing protein | 115941 | 71883.2 | 0.619998129 | 0.6896642 |
| hcp | Hydroxylamine reductase | 128769 | 79926.4 | 0.620695963 | 0.6880413 |
| lptD | LPS-assembly protein LptD | 244469 | 152662 | 0.624463633 | 0.6793105 |
